# The synthesis of deuteriated tri‐*tert*‐butyl phosphine

**DOI:** 10.1002/jlcr.4001

**Published:** 2022-09-05

**Authors:** Lucy C. Brown, Anne McGrogan, Yoan Delavoux, James M. Hogg, John D. Holbrey, H. Q. Nimal Gunaratne, Małgorzata Swadźba‐Kwaśny, James P. Tellam, Sarah E. Youngs

**Affiliations:** ^1^ The QUILL Research Centre, School of Chemistry and Chemical Engineering Queen's University Belfast Belfast UK; ^2^ Department of Chemical Engineering University of Bath Bath UK; ^3^ ISIS Neutron and Muon Source, Rutherford Appleton Laboratory, Harwell Campus Didcot UK

**Keywords:** Grignard, perdeuteriated, perdeuteriated ligands, phosphine, phosphine frustrated Lewis pairs, PtBu3, transition metal catalysis, tri‐tert‐butyl phosphine

## Abstract

The synthesis of deuteriated tri‐*tert*‐butyl phosphine is reported. This synthesis is an adaptation of the known procedure for tri‐*tert*‐butyl phosphine via a Grignard intermediate.

## Graphical abstract

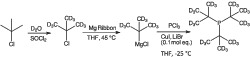

## 1 INTRODUCTION

Tri‐*tert*‐butylphosphine (P^*t*^Bu_3_) is used as a ligand in transition metal catalysis, from common palladium‐promoted cross coupling reactions^1^ to less usual copper‐catalysed [3 + 2] cycloaddition of cyclic ketones to olefins or alkynes.^2^ The combination of Pd(dba)_2_ (dba—dibenzylideneacetone) and P^t^Bu_3_ ligands was used in the amination of various carbon nucleophiles and in the Heck reaction.^3^ Finally, P^*t*^Bu_3_ has been used as a Lewis base component of frustrated Lewis pairs (FLPs), combinations of Lewis acids and bases that are unable to form an adduct due to steric hindrance, but are capable of cooperative catalysis,^4^ in particular metal‐free activation of H_2_ ^5^ and N_2_.^6^

The target of this work was to synthesise, for the first time, fully deuterated [D_27_]tri‐*tert*‐butylphosphine to provide an isotopic contrast for the neutron diffraction studies of the structure of the encounter complexes of FLPs in a benzene solution.^7^

The synthesis of tri‐*tert*‐butyl phosphine (P^t^Bu_3_) is challenging due to the sterically bulky groups surrounding the phosphorus centre. The first attempts to synthesise P^*t*^Bu_3_ via PCl_3_ and a Grignard intermediate, reported in 1967, produced only P^*t*^Bu_2_Cl regardless of the excess of the Grignard reagent used.^8^ Reaction of P(^*t*^Bu)_2_Cl with isopropyl magnesium bromide gave only 22% of the tertiary phosphine, demonstrating that the hindrance resulted directly from the steric encumbrance around the phosphorus centre. Organolithium compounds were found to be more reactive towards P(^*t*^Bu_2_)Cl than Grignard reagents, and tri‐*tert*‐butylphosphine was successfully synthesised from P(^*t*^Bu)_2_Cl by reaction with one equivalent of ^*t*^BuLi, though the overall yields were low (around 17%).

Later, CuI in the presence of LiBr was found to enhance the reactivity of Grignard reagents with P(^*t*^Bu)_2_Cl,^9^ which was used to synthesise P^*t*^Bu_3_ from PCl_3_ ^10^ and from P^*t*^Bu_2_Cl.^11^ In both cases, were prepared at an industrial scale, via the Grignard intermediate, ^*t*^BuMgCl.^12^ Though using PCl_3_ as a starting material is advantageous due to its lower price, it requires cooling to −20°C and additives: 10 mol% of CuI and 20 mol% of LiBr. In contrast, the P^*t*^Bu_2_Cl starting material reacts at near‐ambient temperatures (20–40°C), with much lower demand for additives (1 mol% of CuCl). The challenge in transferring these techniques into the research lab, and onto a small scale, is highlighted in a review by Fleckenstein et al., stating that ‘this reaction appears to require very carefully controlled conditions to produce significant amounts of product’, which favours larger scales.^12^

Building on from various literature procedures for the synthesis of protiated P^*t*^Bu_3_, herein we report, for the first time, a procedure for the synthesis of fully deuterated P^*t*^Bu_3_, achieving yields up to 18.8% at half‐gram scale.

## 2 RESULTS AND DISCUSSION

The first step in the preparation of [D_9_]*tert*‐butyl chloride from commercially available reactants was deuteration of *tert*‐butyl chloride, by treating it with a mixture of D_2_O and thionyl chloride, following a procedure adapted from Prüsse et al.^13^ In contrast to the literature procedure, which is carried out under atmospheric pressure with reflux, it was found more efficient to work under autogenous pressure using a pressure‐resistant sealed tube (90% vs. 95% isotopic enrichment was achieved after two cycles, assessed by ^1^H NMR spectroscopy). Furthermore, in order to complete the removal of SO_2_ produced in the first step, it was necessary to wash the product with dichloromethane (DCM). Unfortunately, the product was found to boil slightly above 40°C, which resulted in some product being removed with DCM (bp 39.6°C) during the purification step. Analytical data are reported in Supporting Information (Schemes S1–S5).

**SCHEME 1:**
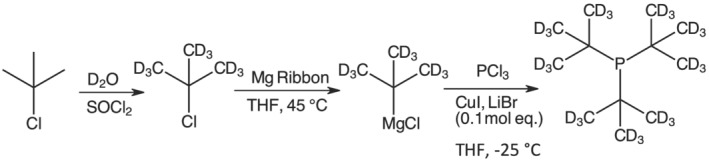
The synthesis of [D_27_]P^t^Bu_3_ via a Grignard intermediate, [D_9_]^t^BuMgCl

The procedure for the synthesis of [D_27_]tris‐*tert*‐butyl phosphine (Scheme 1) was developed by combining aspects of the methodology developed for its protiated analogue.^10^ The need to develop a dedicated procedure for the synthesis of [D_27_]P^*t*^Bu_3_ arose from a strong kinetic isotope effect observed.

The first step of the reaction was the formation of [D_9_]^t^BuMgCl. This was encouraged by activating magnesium ribbon by brushing it with sandpaper to create a rough surface and cutting it into small flakes. Furthermore, the magnesium flakes were stirred overnight under an inert atmosphere, to increase the available exposed magnesium sites. The Grignard formation was very exothermic, which made THF (bp 66°C) preferable to the more volatile diethyl ether. Gentle heating over a heating mantle was essential to overcome activation energy and to initiate the reaction. Once initiated, however, the reaction required cooling in an ice bath to prevent the loss of reactants by evaporation (see the Section 3 for a detailed addition procedure). Achieving the balance between heating and cooling was the key to achieving high conversions to the Grignard reagent in the solution. The generation of the Grignard intermediate was additionally promoted by the addition of a crystal of iodine and a few drops of dibromoethane. Both additives have been reported to promote Grignard intermediate generation in this reaction.^14^

**FIGURE 1:**
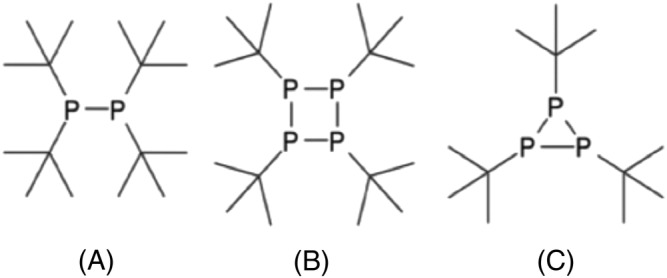
Side products generated in the synthesis of P^t^Bu_3_

In the second step (Scheme 1), the main challenge lays in minimising radical side reactions, which lead to the formation of less sterically hindered diphosphane and cyclic products (Figure 1).^15^, ^16^

Because the bulky *tert*‐butyl group in ^*t*^BuPCl_2_ introduces steric hindrance to the phosphorus centre, the formation of P‐P bonds is favoured over the second and third *tert*‐butyl substituent, which favours selectivity towards the undesired side‐products (Figure 1), rather than P(^t^Bu)_3_ ^15^; note highly selective synthesis of cyclotetraphosphine (Figure 1B) from P^*t*^BuCl_2_ with bis‐imidazoline.^16^

In order to enhance selectivity to P^*t*^Bu_3_, it was important to remove any potential reducing agents, inhibit radical reaction pathways and to use large excess of the Grignard reagent (^*t*^BuMgCl), while keeping PCl_3_ in high dilution. To eliminate contamination with traces of magnesium ribbon (the main source of a potential reducing agent), the addition of the Grignard reagent to the solution of PCl_3_ was performed via a cannula filter. To inhibit radical pathways, following the work reported in patents to Bayer and Hokko,^10^, ^11^, ^12^ lithium bromide and copper(I) iodide were added to the PCl_3_ solution before the Grignard reagent was added, at 10 mol% loading of both salts. Increasing the quantity of lithium bromide from 10 to 20 mol%, as reported in the patent,^10^ did not have a significant impact on yield.

**FIGURE 2:**
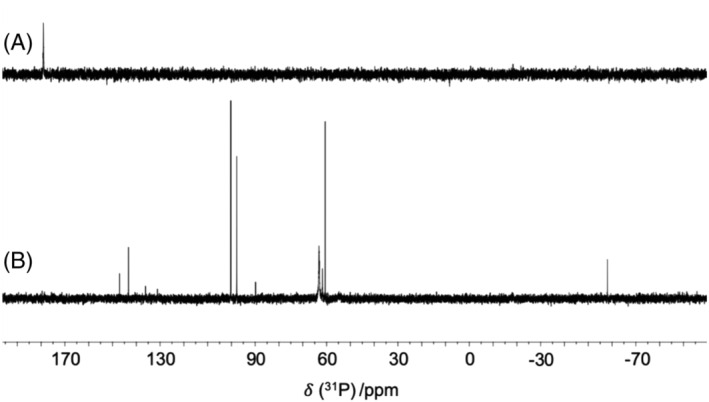
A comparison of ^31^P NMR spectra (A) in the presence of copper(I) iodide and lithium bromide at 10% mol ratio and (B) in the absence of these salts

To examine the influence of these salts on the reaction selectivity, an excess of PCl_3_ was reacted with ^*t*^BuMgCl in the presence, and absence, of 10 mol% of CuI and LiBr (Figure 2, ^31^P NMR spectra A and B, respectively). When both salts were present (Figure 2A), dichloro(*tert*‐butyl)phosphine (PCl_2_^*t*^Bu) was generated as a single product (*δ*_31P_ = 190.0 ppm),^17^ but when CuI and LiBr were not present, radical reactions were not inhibited, resulting in a reaction mixture containing a large number of ^31^P NMR signals (Figure 2B). All these resonances have *δ*_31P_ shifts that are more shielded than that of P^*t*^BuCl_2_, in addition to a peak at −58 ppm, which represents the cyclotetraphosphine (Figure 1B).^18^ Thereby, convincing evidence has been obtained that the desired reaction pathway is achieved by the introduction of small quantities of CuI and LiBr.

**FIGURE 3:**
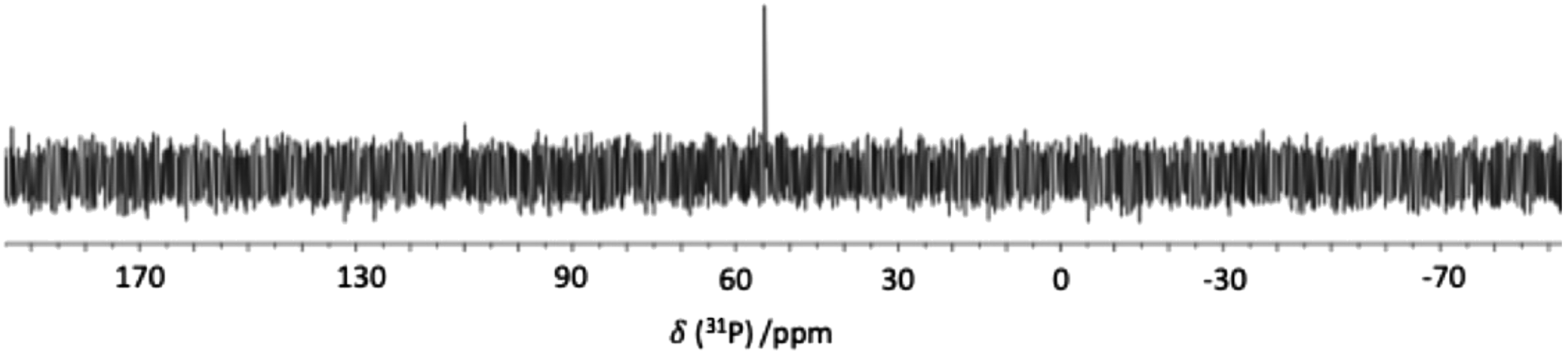
The ^31^P NMR spectrum of the product [D_27_]tert‐butyl chloride

Following synthesis, P^*t*^Bu_3_ was purified by removing the reaction solvents under reduced pressure, dissolving the products in pentane and washing the solution with degassed water. The organic phase was then dried and, upon removal of solvent, P^*t*^Bu_3_ was obtained as a colourless, crystalline solid. The structure of the product was confirmed by ^31^P NMR spectroscopy (Figure 3), combined with ^13^C NMR spectroscopy and mass spectrometry, and was consistent with [D_27_]P^*t*^Bu_3_.

Analytical data are reported in Supporting Information (Schemes S4–S7). ESI‐MS show molecular peak at 230.36, consistent with [H_1_D_26_]P^*t*^Bu_3_, which corresponds well to the ^2^ *δ* = 95% value recorded from 1H q‐NMR for the deuteriated starting material, [D_9_]^*t*^BuCl. Considering the pyrophoric nature of this phosphine, it was crucial to maintain full air exclusion and ensure that all solvents were well degassed; failure to do so resulted in rapid formation of oxidation products.

In conclusion, syntheses of a sterically hindered phosphine, P^*t*^Bu_3_, in its perdeuterated form, have been reported for the first time, with a yield that matches those reported for the protiated equivalent. A detailed synthetic procedure, described in this contribution, contrasts brief reports found elsewhere and should be particularly useful to those that do not typically work with phosphine chemistry. It is hoped that it will enable and encourage further neutron scattering studies by colleagues interested in FLP chemistry. Finally, the procedure described in this contribution can be relatively easily adapted to synthesise other labelled or unlabelled, trialkylphosphines.

## 3 EXPERIMENTAL

### 3.1 General

*Tert‐*butyl chloride was synthesised at the Deuteration Facility at ISIS Neutron and Muon Source, STFC Rutherford Appleton Laboratory in Oxfordshire, UK. Phosphorus trichloride (99%), magnesium ribbon (>99.5%), copper(I) iodide (>99.5%) and lithium bromide (>99%) were purchased from Sigma Aldrich and used as received. The glovebox used in this work was MBraun Labmaster dp, operating typically under argon, at or below 0.6 ppm of H_2_O and O_2_. ^13^C and ^31^P NMR spectra were recorded on a Bruker Avance DPX 400 MHz spectrometer. Electrospray mass spectrometry measurements were performed using a Waters LCT Premier mass spectrometer.

### 3.2 Syntheses of [D_9_]*tert*‐butyl chloride

D_2_O (100 cm^3^) was placed in a thick‐walled glass tube (250 cm^3^) equipped with a stirring bar and a screw top with PTFE seal. The tube was placed in an ice bath (0°C) standing on a heater‐stirrer, and D_2_O was allowed to cool, while stirring vigorously. Subsequently, the screw top was opened, and thionyl chloride (40 cm^3^, 65.5 g, 0.551 mmol) was added dropwise, followed by the addition of *tert*‐butyl chloride (42 cm^3^, 33.7 g, 0.386 mmol). Then, the tube was sealed with the screw top, the ice bath was replaced with an oil bath and the mixture was left to react (110°C, 24 h, vigorous stirring). Afterwards, reaction mixture was cooled to ambient temperature in air and subsequently to 0°C, resulting in phase separation. The aqueous phase (bottom layer) was then decanted and isolated from the organic phase (top layer), and the organic phase was sampled for quantitative NMR (qNMR) spectroscopy, with DCM as internal standard (approximately 20 mg product and approximately 40 mg DCM were weighed accurately, using a 5‐digit balance, into 500 μl of CDCl_3_); the relative integration of the NMR peak for DCM (2H) and tert‐butyl chloride (9H) resulted in a isotopic substitution of ^2^ *δ* = 77%. The same procedure was repeated for another cycle, using fresh D_2_O and thionyl chloride, resulting in ^2^ *δ* = 95%. Natural abundance of ^1^H isotope is 99.97%, and the error of the qNMR method used in this was found to be approximately ±0.5%; therefore, the natural abundance is approximated as 100%, and isotopic enrichment (^2^ *δ*) is reported to the nearest integer value.

The organic phase was then dissolved in dichloromethane (40 cm^3^) and washed several times with water (20 cm^3^). The sample was degassed to remove remaining traces of sulphur dioxide, and dichloromethane was distilled at atmospheric pressure using a Vigreux column. The product was then dried by distillation resulting on a colourless liquid, 16.0 g (yield 41%) and was analysed by qNMR (44.0 mg DCM, 28.2 mg [D_9_]^*t*^BuCl).

^1^H‐qNMR (400 mHz, CDCl_3_) δ 5.30 ppm (s, *I* = 1, 2H, C*H*_*2*_Cl_2_), δ 1.57 ppm (m, *I* = 0.12, 9H, (C*H*_*3*_)_3_C‐Cl).

### 3.3 Syntheses of [D_27_]tri‐*tert*‐butyl phosphine

Magnesium ribbon (1.80 g, 74.1 mmol) was activated inside an argon filled glovebox by scratching with sandpaper, then cut into flakes and transferred into an oven‐dried two‐necked round‐bottomed flask (100 cm^3^) equipped with a septum, a tap and a stirring bar. The flask was removed from the glovebox, attached to an argon Schlenk line and placed on a heater stirrer equipped with an aluminium heating mantle. The magnesium was stirred overnight at ambient temperature to further activate the magnesium. Then, dry tetrahydrofuran (20 cm^3^) was added, followed by a crystal of iodine, and the flask was heated to 40°C. Subsequently, a small portion (about 20 vol. %) of *tert*‐butyl chloride‐*d*_9_ (6.27 g, 61.7 mmol) was added to initiate the reaction, with the solution turning green, before the remainder was added dropwise, over the course of 5 min. Subsequently, a small amount of dibromoethane (0.10 g, 0.57 mmol) was added dropwise to encourage the reaction, which was then allowed to proceed (40°C, overnight), before being cooled back to room temperature.

In another oven‐dried two‐necked round‐bottomed flask (100 cm^3^), equipped with a stirring bar and connected to an argon Schlenk line, a phosphorus trichloride (1.70 g, 12.35 mmol), with lithium bromide (0.107 g, 1.24 mmol) and copper(I) iodide (0.235 g, 1.24 mmol), was added to degassed, dry THF (15 cm^3^). The flask was placed on a heater stirrer equipped with an acetone‐dry ice bath (−78°C), and the mixture was allowed to cool with vigorous stirring.

The solution of the Grignard reagent was transferred via a cannula filter into the PCl_3_ solution and stirred at −78°C. The dry ice‐acetone bath was then removed, and the reaction mixture was brought to ambient temperature and left to react for a further 2 h with vigorous stirring on reaching this temperature. The solvent was removed under reduced pressure (25°C, 10^‐2^ bar), and the product was dissolved in pentane (25 cm^3^). Degassed water (25 cm^3^) was subsequently added, and the flask was vigorously shaken by hand, and the organic layer was removed via cannula transfer into an oven dried flask (100 cm^3^) and again washed with degassed water (25.0 cm^3^). This was transferred via cannula into an oven‐dried flask (100 cm^3^). Finally, the organic phase was dried using sodium sulphate, and the liquid phase was transferred via cannula filtration into an oven‐dried flask (100 cm^3^). The solvent was removed under reduced pressure in an ice bath (0°C, 10^‐2^ bar), to give a colourless crystalline solid, 0.469 g (18.8% yield).

^31^P NMR (162 MHz, benzene‐*d*_6_) δ 56.0 (s); ^13^C {^1^H} (101 MHz, benzene‐*d*_6_) *δ* 37.0 (d); J_C‐P_ = 51.3 Hz, *δ* 26.8 (m); *m*/*z* (‐ve ion electrospray) [M‐H]‐ C_12_D_27_P required 230.3631, found 230.3623.

## Supporting information


**Scheme S1.** ^1^H NMR spectrum (400.13 Hz, 298 K) of [D_9_]^t^BuCl in [D_1_]trichloromethane, with dichloromethane internal standard.
Scheme S2. ^2^H NMR spectrum (61.42 Hz, 298 K) of [D_9_]^t^BuCl in [D_1_]trichloromethane.
Scheme S3. ^13^C NMR spectrum (100.62 Hz, 298 K) of [D_9_]^t^BuCl in [D_1_]trichloromethane.
Scheme S4. ^13^C NMR spectrum (100.56 Hz, 298.9 K) of [D_27_]^Pt^Bu_3_ in [D_6_]benzene. “
Scheme S5. ^31^P NMR spectrum (161.98 Hz, 300 K) of [D_27_]^Pt^Bu_3_ in [D6]benzene.
Scheme S6. ESI‐MS pattern for [D_27_]^Pt^Bu_3_

## Data Availability

Data available on request from the authors.
