# Supplementary material for: The synthesis of deuteriated tri‐tert‐butyl phosphine
Source: J Labelled Comp Radiopharm. 2022 Sep 5;65(13):338–42. doi: 10.1002/jlcr.4001 (PMC9826328; doi:10.1002/jlcr.4001)
Supplement: Supplementary file 1 — Scheme S1. 1H NMR spectrum (400.13 Hz, 298 K) of [D9]tBuCl in [D1]trichloromethane, with dichloromethane internal standard. Scheme S2. 2H NMR spectrum (61.42 Hz, 298 K) of [D9]tBuCl in [D1]trichloromethane. Scheme S3. 13C NMR spectrum (100.62 Hz, 298 K) of [D9]tBuCl in [D1]trichloromethane. Scheme S4. 13C NMR spectrum (100.56 Hz, 298.9 K) of [D27]PtBu3 in [D6]benzene. “ Scheme S5. 31P NMR spectrum (161.98 Hz, 300 K) of [D27]PtBu3 in [D6]benzene. Scheme S6. ESI‐MS pattern for [D27]PtBu3 [file JLCR-65-338-s001.pdf]

The synthesis perdeuteriated tri-*tert*-butyl phosphine

Lucy C. Brown,[a] Anne McGrogan,[a] Yoan Delavoux,[a] James M. Hogg,[a,b] John D. Holbrey,[a] H. Q. Nimal Gunaratne,[a] Małgorzata Swadźba-Kwaśny,*[a] James P. Tellam[c] and Sarah E. Youngs*[c]

Supplementary Information

Analytical data for [D_9]*tert*-butyl chloride are shown in Figure 1-SI – Figure 3-SI.

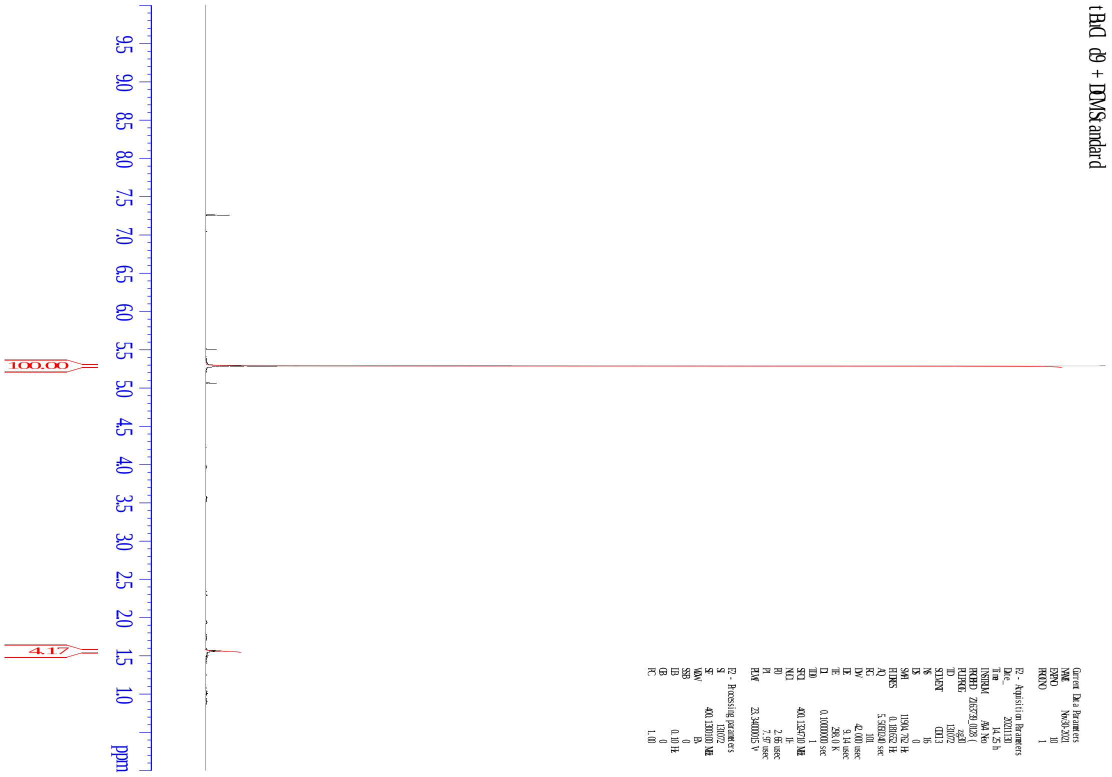

Scheme 1-SI. ^{1}H NMR spectrum (400.13 Hz, 298 K) of [D_9]^{t}BuCl in [D_1]trichloromethane, with dichloromethane internal standard.

.

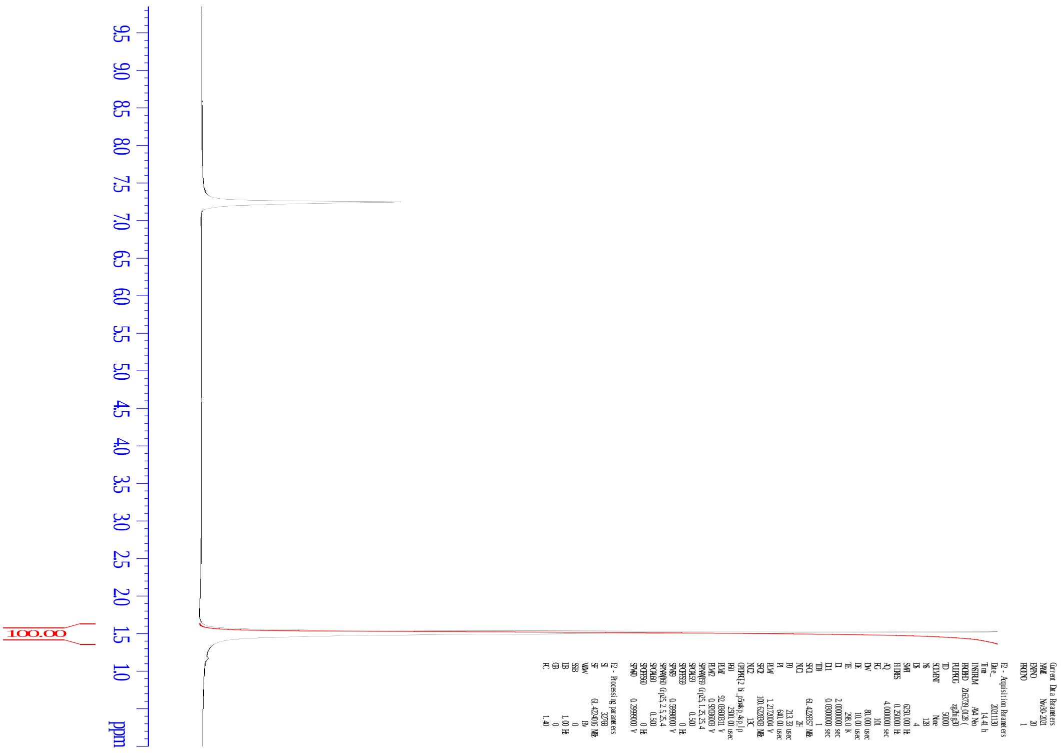

Scheme 2-SI. ^{2}H NMR spectrum (61.42 Hz, 298 K) of $[D_9]^{t}BuCl$ in $[D_1]$trichloromethane.

.

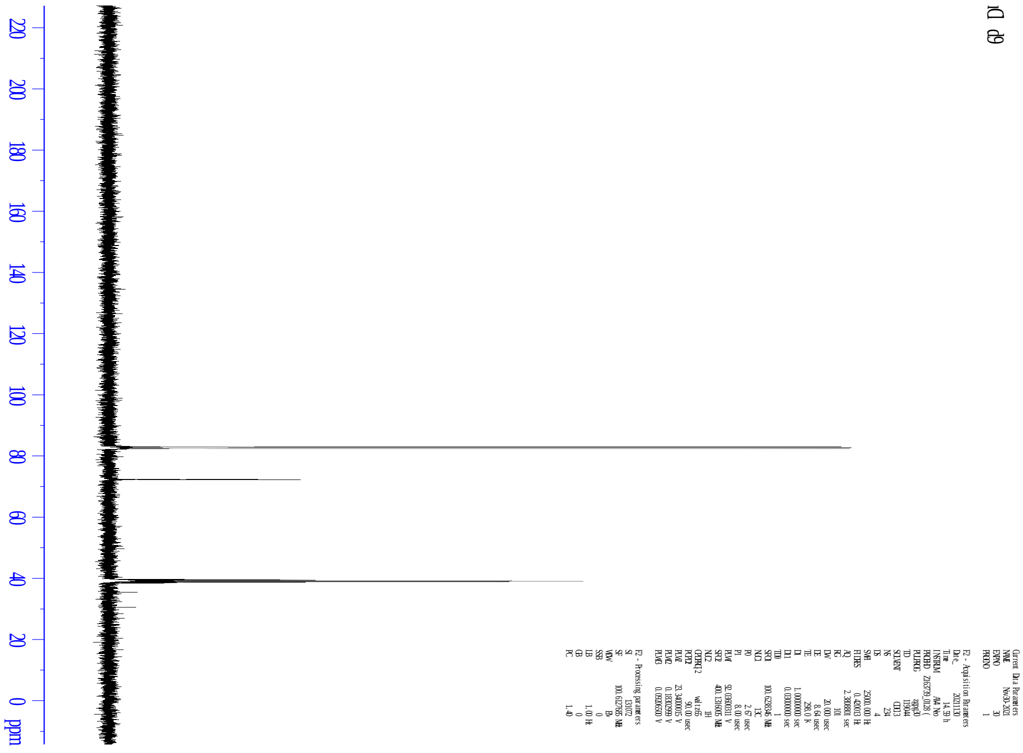

Scheme 3-SI. ^{13}C NMR spectrum (100.62 Hz, 298 K) of $[D_9]^{t}BuCl$ in $[D_1]$trichloromethane.

Analytical data for [D_{27}]tris-*tert*-butyl phosphine are shown in Figure 4-SI – Figure 7-SI.

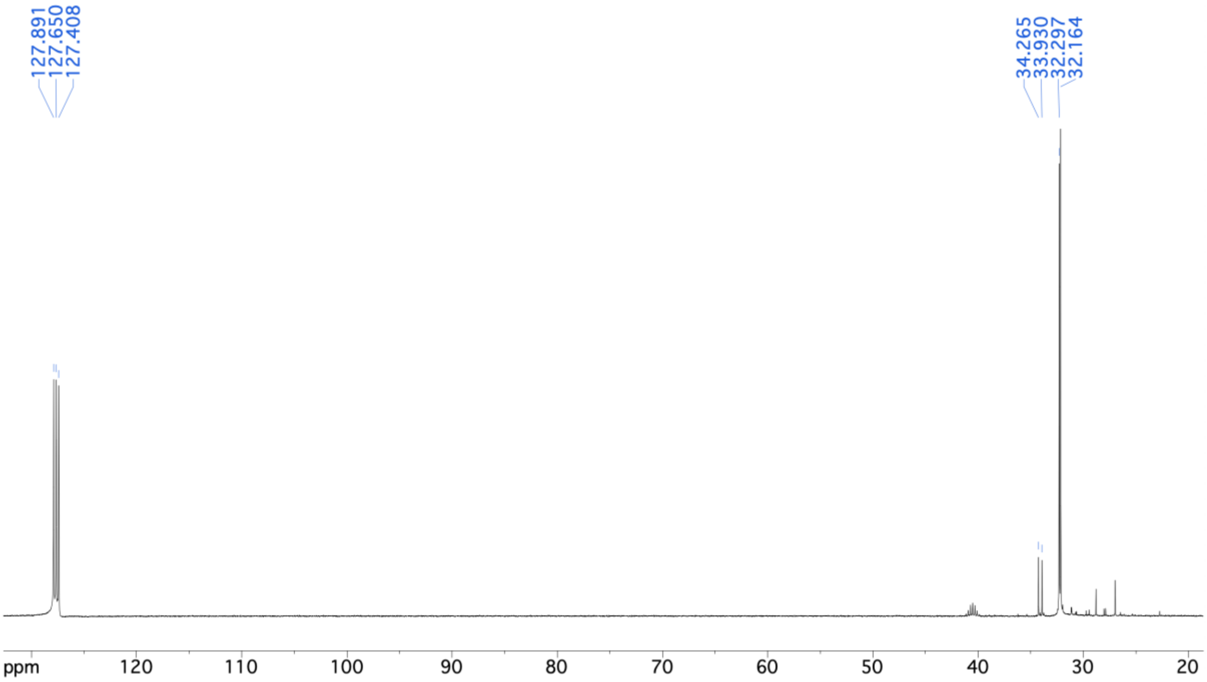

Scheme 4-SI. ^{13}C NMR spectrum (100.56 Hz, 298.9 K) of [D_{27}]P^{t}Bu$_3$ in [D_6]benzene.

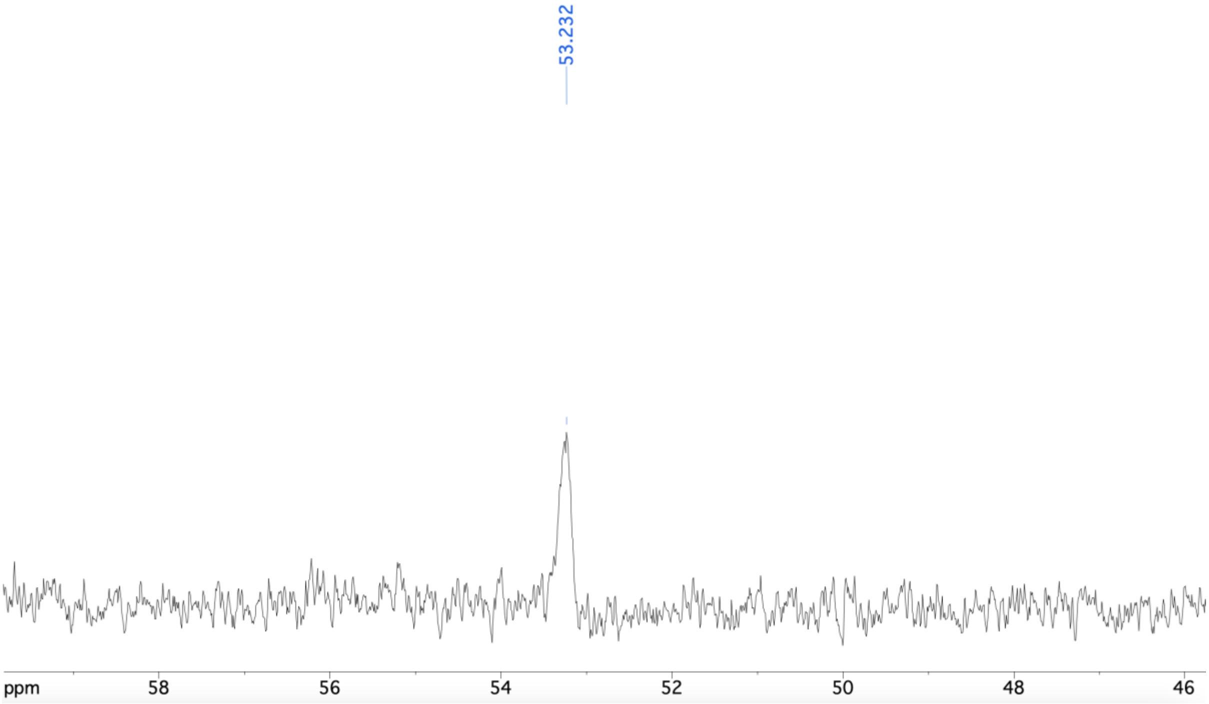

Scheme 5-SI. ^{31}P NMR spectrum (161.98 Hz, 300 K) of [D_{27}]P^{t}Bu$_3$ in [D_6]benzene.

Single Mass Analysis

Tolerance = 5.0 PPM / DBE: min = -1.5, max = 50.0
Element prediction: Off
Number of isotope peaks used for i-FIT = 4

Monoisotopic Mass, Even Electron Ions
1571 formula(e) evaluated with 1 results within limits (all results (up to 1000) for each mass)
Elements Used:
C: 0-120 P: 1-1 1H: 0-180 2H: 0-180

LBA-124
asep__07JAUG_2018_31 6 (0.283) Cm (5:15) 1: TOF MS ES+
1.45e+004

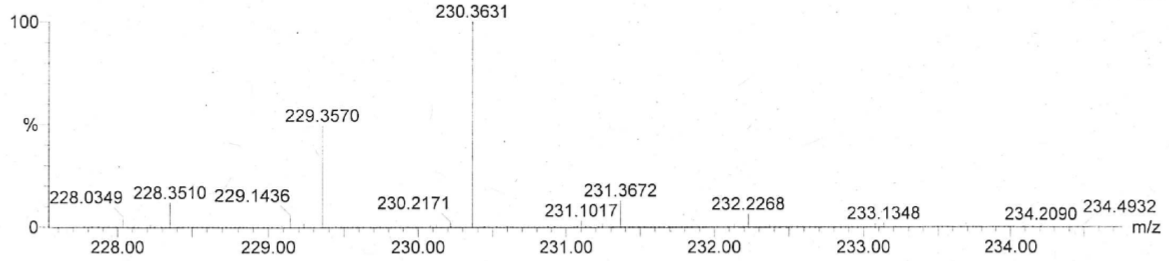

Minimum:				-1.5		
Maximum:		5.0	5.0	50.0		
Mass	Calc. Mass	mDa	PPM	DBE	i-FIT	Formula
230.3631	230.3623	0.8	3.5	-0.5	6.6	C12 P 1H 2H27

Scheme 6-SI. ESI-MS pattern for $[D_{27}]P^{t}Bu_3$.
